# Identification des risques professionnels dans l'industrie textile en République Démocratique du Congo

**DOI:** 10.11604/pamj.2014.19.373.4186

**Published:** 2014-12-11

**Authors:** Panda Lukongo Kitronza

**Affiliations:** 1 Ecole de Santé Publique, Faculté de Médecine, Université de Liège, Belgique; 2 Faculté de Médecine, Université de Kisangani, Congo RD

**Keywords:** Risque, coton, textile, santé, travail, Congo, risk, cotton, textile, health, work, Congo

## Abstract

**Introduction:**

Le but de cette étude est de mettre en évidence les facteurs de risques professionnels liés aux conditions de travail.

**Méthodes:**

Cette étude qualitative basée sur les entretiens de groupe a été réalisée par une équipe pluridisciplinaire dans l'industrie textile de la région du Est de la RDC; comprenant un médecin de travail, un médecin de santé publique, un toxicologue, deux infirmiers du centre hospitalier de l'usine, un représentant du comité d'hygiène et un technicien de prévention. La démarche méthodologique a consisté en des entretiens en groupe, des observations et visites guidées de lieux de travail de l'entreprise.

**Résultats:**

Dans la culture du coton, les effets d'une forte exposition aux pesticides peuvent entraîner des intoxications aiguës, chroniques et voire le décès. Les autres risques sont les accidents de travail, les maladies professionnelles, les troubles psychologiques. Dans l'industrie, les travailleurs sont exposés aux risques liés à l'empoussiérage des fibres de coton, aux facteurs des risques traumatiques, physiques (bruits, vibration) et chimiques (acides forts, bases fortes, solvants et colorants minéraux), ainsi qu'aux risques psychosociaux. La pollution de l'environnement et l’écotoxicité inhérente à ces activités restent l'effet de l'usage des grandes quantités d'intrants agricoles, engrais et produits phytosanitaires.

**Conclusion:**

Cette étude a permis de mettre en évidence les différents facteurs de risques auxquelles sont soumis les travailleurs textiles; ainsi que les risques environnementaux liés à cette activité. Cela est de nature à permettre la mise sur pied d'une stratégie efficace de prévention et de protection des travailleurs.

## Introduction

La protection des travailleurs contre les risques professionnels est l'un des objectifs de l'organisation internationale du travail, car, sécurité et santé au travail sont non seulement indispensables au travail décent mais constituent aussi un facteur important de la croissance économique, de la productivité et du développement [1, 2]. Le Bureau International du Travail estime que les risques générateurs de mauvaise santé au travail sont plus élevés dans les pays en développement, au rang desquels figure la République Démocratique du Congo (RDC) et dans les pays nouvellement industrialisés [3, 4]. La plupart des entreprises dans ces pays semblent être moins protégées par les lois sur la sécurité et la santé au travail [1, 3, 4]. Les conditions d'emploi des travailleurs,sont en général précaires et méritent une analyse focalisée sur les facteurs de risques [5]. L'activité textile recouvre aujourd'hui toute une série des procédés qui constitue la chaîne textile [6]. Cette chaîne comprend la production du coton brut; l’égrainage; la filature; le tissage et la bonneterie; le finissage; la confection; la vente, l'utilisation et l’élimination. Il s'agit d'une activité qui met en péril le bien être des travailleurs, en raison du grand nombre de risques (bruit, chaleur, poussières etc.) et des contraintes physiques (mouvements répétitifs, port des charges lourdes, postures contraignantes etc.) et psycho-organisationnelles (insécurité d'emploi, charge et rythme de travail, horaire atypique et irrégulier etc.). Dans cette période de crise socio-économique intense en RDC, le secteur de l'industrie textile constitue une des principales activités pourvoyeuses d'emploi au pays. Les travailleurs semblent, pour besoin d'emploi, exposés à nombreux risques de sécurité et de santé au travail au mépris de normes en vigueur. Le but de cette étude est donc d'identifier les différents facteurs de risques dans l'industrie textile. Elle est de nature à contribuer à l'amélioration du bien-être au travail dans cette région en faisant le point de connaissance et en réunissant les informations sur les risques professionnels liés à cette activité dans la province orientale de la RDC. Les études approfondies seront diligentées enfin de mettre en exergue les problèmes de santé des travailleurs dans cette entreprise. Ce travail constitue une nouvelle approche de la santé au travail dans le secteur de l'industrie textile en RDC. Son intérêt majeur réside dans les implications et applications qui pourraient en découler en termes de la prise en charge des problèmes de santé existants, de la prévention, de la protection et de la promotion de la santé et du bien-être des travailleurs.

## Méthodes

Cette étude qualitative à visée descriptive et exploratoire est basée sur des entretiens des groupes avec des travailleurs textiles. Elle s′est déroulée de 10 mai à 30 juin 2009 au sein du complexe agro-industriel, qui est une usine textile située dans la province Nord-est de la RDC. L'usine employait au moment de l'enquête 987 agents, selon la liste établie par la direction des ressources humaines. La méthode des entretiens est une méthode qualitative de recherche sociale qui tend à faire ressortir toutes les opinions sur un thème spécifique, afin de mieux comprendre certains phénomènes sociaux [7]. Elle dégage alors une évaluation qualitative qui pourrait aider à l’élaboration d'outils quantitatifs pouvant mesurer les différents concepts [8]. L’étude a été conduite par une équipe pluridisciplinaire comprenant un médecin de travail et responsable de l’étude, un médecin de santé publique, un toxicologue, un technicien de prévention, deux infirmiers du centre hospitalier de l'usine et un représentant du comité d'hygiène et des conditions de travail. Ils ont été formés sur le guide d'entretien de l’étude. L'enquête a été réalisée après avoir expliqué le but de cette étude à la hiérarchie et aux travailleurs. A été retenu à l’étude, le travailleur avec au moins cinq ans d'expérience et ayant accepté librement de participer. Étant donné le caractère pilote et exploratoire de cette étude, le nombre de groupes a été limité à sept. Tenant compte des modalités pratiques; 100 personnes ont été retenues parmi les travailleurs par un échantillonnage raisonné de convenance. L'objectif des enquêtes qualitatives étant de comprendre des situations, et non pas d'estimer des valeurs d'une population d'enquête, le nombre de sujets a été choisi de façon raisonnée. Il s'agit de la sélection raisonnée de personnes choisies pour leur diversité de façon à prendre en compte les situations sociales les plus différents possibles, au sein de la population étudiée [9, 10].

Les discussions étaient enregistrées/notées systématiquement après un consentement éclairé des participants. La démarche méthodologique a consisté en: des entretiens avec les délégués du personnel et les organisations syndicales de l'entreprise, 2 groupes de 10; des entretiens avec les travailleurs ouvriers et employés, 5 groupes de 12; des entretiens avec les membres du comité d'hygiène, santé et des conditions de travail, 2 groupes de 10; des observations lors des visites guidées des postes de travail.

Le guide d'entretien et observations contenait les aspects thématiques ci-après: les expositions physiques (bruit, chaleur, vibration, traumatisme etc.), chimiques (exposition acide, base, peinture etc.), biologiques (contact agent biologique, piqûres insectes et serpents venimeux), ainsi les facteurs des risques psychosociaux (stress) au niveau de la culture du coton; les expositions au sein de la chaîne textile; les impacts environnementaux liés à l'activité textile.

Les entretiens étaient animés par le médecin responsable de l'enquête, assisté par les autres membres de l’équipe. Les observations et les entretiens ont été faits en respectant l'anonymat et enregistrés/notés intégralement. Dans un premier temps, nous avons fait l'inventaire de toutes les informations recueillies, lesquelles ont été ensuite transcrites. L'analyse de contenu a été effectuée à partir des transcriptions et des notes terrain prises par les chercheurs, eu égard à l'approche ouverte et inductive des généralisations des données [11, 12].

## Résultats

Les informations recueillies nous ont permis de faire ressortir toute la typologie des risques auxquels les travailleurs du secteur sont exposés. Nos résultats portent respectivement sur les risques professionnels dans la production cotonnière et dans l'industrie de transformation du coton. Nous aborderons afin les risques environnementaux liés à cette activité tels signalés par les enquêtés. Par manque des données chiffrées, il a été difficile de déterminer les pourcentages des affections signalées par les enquêtés.

**Dans la production et la campagne de commercialisation du coton:** il ressort à partir des entretiens réalisés que les travailleurs sont victimes de nombreux accidents, maladies et intoxications. De manière globale, les risques suivants ont été majoritairement inventoriés. Les risque toxiques par usage des produits phytosanitaires, avec comme dommage les intoxications aigues. Les cultivateurs déclarent utiliser souvent les produits phytosanitaires industriels (pesticides insecticides et herbicides) et parfois les engrais. L'utilisation des produits phytosanitaires artisanaux faits de préparations magistrales contenant le mélange pesticides, engrais et gasoil ont été également signalés. Les cas d'intoxications aigues ont été signalés tandis que les chroniques n’étaient pas signalés. L'utilisation domestique de ces produits contre les vecteurs du paludisme reste fréquente. Certains se servent des insecticides pour tentative des suicides. Dans les risques traumatiques liés à la mécanisation progressive de l'agriculture et traduite souvent par les accidents de travail (environ 60% des cas), on note les blessures légères ou graves, les contusions, les entorses, les fractures, et rarement les amputations. Les troubles musculosquelettiques consécutifs aux manutentions, au port des charges lourdes et aux positions contraignantes ont été également signalés. Les expositions à des températures extrêmes sont quasi permanentes. Le plus souvent, il s'agit d'ambiances très chaudes et humides, conditions propices aux coups de chaleur et aux insolations. Les risques des troubles psychologiques favorisés par les inondations des champs et les ravages de ceux-ci par les insectes ravageurs et les rongeurs, entrainant ainsi les atteintes anxio-dépressives et le stress. Dans les facteurs de risques biologiques, les risques infectieux liés à la présence de microorganismes pouvant déclencher le tétanos, la brucellose par contact avec les animaux contaminés (ovins et caprins). Les risques liés aux facteurs socio-organisationnels du travail dont les facteurs favorisants évoqués sont l'importante charge du travail, le port des charges lourdes, le travail répétitif, en équipe (rivalité), l'absence de formation et/ou d'information, le manque ou insuffisances des EPI, la prise en charge médicale insuffisante ou inexistante. Les risques liés aux morsures des serpents et autres insectes venimeux dans les champs avec possibilité d'envenimation.

**Dans l'industrie de transformation du coton:** les différents procédés de fabrication présentent des risques majeurs pour la santé des travailleurs dont les plus significatifs signalés et répertoriés. Les risques d'accidents de travail sous formes de blessures, fractures, entorses, amputations, brulures etc. Les risques liés à l'empoussiérage aux fibres du coton traduits par les atteintes broncho-pulmonaires (toux, bronchite, pneumonie etc.). Les risques liés aux bruits, à la chaleur, aux vibrations, à la manutention de lourdes charges, aux mouvements répétitifs et aux positions contraignantes. Ils se caractérisent par les troubles d'audition, les déshydrations et coup de chaleur, les TMS. Les risques chimiques par exposition aux acides, bases, solvants et colorants minéraux caractérisés par les atteintes cutanéo-muqueuses, les irritations, les allergies et parfois les intoxications. Les risques liés aux problèmes psycho-sociaux et organisationnels, à la base de la dépression, de stress et à la longue de burn out.

**Risques environnementaux:**outre l'atteinte à la santé humaine, les risques environnementaux liés à l'activité textile ont été relevés. Il s'agit de de la contamination des eaux de surface et des eaux souterraines par les pesticides et de la pollution atmosphérique par les eaux usées, l'empoussiérage du milieu environnant, les déchets et les odeurs dans les lieux résidentiels voisins.

## Discussion

Dans la production cotonnière, les pesticides représentent les agents étiologiques des principaux problèmes de santé dans les populations des travailleurs [6, 13, 14]. Notre enquête a relevé que tous les producteurs du coton traitent leur champ avec les pesticides, les plus utilisés étant les insecticides et les herbicides. Face aux insuffisances de la réglementation en matière d'importation, de vente, de détention et de distribution des pesticides dans les pays en développement [13], observe sur terrain la circulation de nombreux pesticides non agrées par l'institut national d’étude et recherche agronomique;des préparations magistrales des produits des pesticides, d'engrais et gasoil par les exploitants en plus de l'utilisation abusive des produits. Ces observations rejoignent celles des études de l'OMS [15]. Pour cette activité, nous avons constaté que les travailleurs n'utilisent presque pas les équipements de protections requis, souvent par faute des moyens, par négligence ou par manque d'information et de formation sur la nature des produits et les conditions d'utilisation. Tous ces facteurs pourraient expliquer les cas d'intoxication constatés dans la culture du coton. Nos observations sont soutenues par la littérature [16–19] qui soutient que l'atteinte à la santé par les pesticides est fonction de plusieurs critères dont la toxicité du produit, la durée et le nombre d'application, l’état de santé de travailleur, les conditions atmosphériques pendant l'application l'utilisation et la disponibilité ou non d’équipement de protection, l'insuffisance de l'information et de la sensibilisation sur les risques, l'insuffisance des règles d'hygiène au travail et la faiblesse de la législation. La pénétration de ces substances dans l'organisme s'effectue principalement par la voie cutanée, mais aussi par la voie respiratoire et la voie digestive. Plusieurs études soulignent que, très souvent, l'exposition est réalisée par toutes ces voies [11, 13]. Dans notre étude, plusieurs cas d'intoxication aigué ont été reportés et ils ont été pris en charge dans les hôpitaux de la place. Par manque d'un système adéquat et opérant d'enregistrement des cas, nous n'avons pas l'occasion d'en estimer la prévalence. Ceci rejoint le constat de plusieurs études faites dans les pays en développement [4, 12, 13]. Les études de Damalas CA et Michael C.R soutiennent que les effets immédiats d'une forte exposition aux pesticides peuvent revêtir des formes variées allant des atteintes bénignes à type d'urticaires, de vertige, d’épistaxis à des atteintes graves comportant des troubles respiratoires et neurologiques entraînant souvent le décès [20, 21]. Certaines tentatives de suicides liés aux pesticides dans le cadre des intoxications aiguës ont été relevées dans notre enquête. Souvent, les intoxications aigues sont souvent dues à une mauvaise manipulation du produit ou à des tentatives des suicides selon certaines études faites dans ce domaine [11, 22, 23]. Quant aux intoxications chroniques, elles restent souvent méconnues du fait de la survenue tardive des symptômes, du manque de suivi médical lié à la défaillance du système d’évaluation de la santé et à l'absence d'un système opérant des déclarations des cas aigus ou chroniques. Plusieurs études mettent en évidence que la toxicité par exposition prolongée peut induire des cancers, favoriser l'apparition d'effets mutagènes, d'effets tératogènes, des modifications fonctionnelles du système endocrinien, du système immunitaire, des fonctions du foie, du rein, d'autres organes et des réactions d'hypersensibilité [11, 15, 16]. L'utilisation des pesticides à domicile pour lutter contre certains vecteurs (paludisme, fièvre typhoïde) sème l'ambiguïté entre l'intoxication professionnelle et domestique. Cette utilisation abusive par les gens mal formés et informés accroitrait les risques d'intoxication aiguée et de suicide. Les études de l'OMS et celles de Nalwanga et al. confirment nos observations dans ce secteur [4, 24, 25]. Dans notre étude, les cas d'accidents reportés ont été soignés dans les hôpitaux mais non enregistrés. Il était cependant difficile de relever les cas des maladies professionnelles. Les accidents de travail seraient consécutifs à une mécanisation progressive de l'agriculture, comme cela est évoqué dans plusieurs autres études [21, 26, 27]. Les maladies professionnelles quant à elles, sont souvent méconnues et tardives. Elles sont liées à l'empoussiérage et aux facteurs physiques (les bruits, les vibrations, les manutentions de charges lourdes, les contraintes posturales, les mouvements répétitifs etc.). Plusieurs études faites ailleurs évoquent cette situation [23–25, 28, 29]. Les troubles psychosociaux signalés seraient en rapport avec certaines catastrophes naturelles (inondation des champs, attaque massive par insectes ravageurs et des rongeurs), auxquelles vient s'ajouter la mauvaise organisation du travail. Nos observations sont corroborées par celles de plusieurs autres recherches faites dans ce secteur [20, 23, 24, 30].

Dans l'industrie de transformation du coton, les travailleurs sont exposés à différents types de risques. Les facteurs des risques traumatiques sont consécutifs aux machines et engins de transport, aux chutes, aux incendies et combustions, à l'environnement du milieu de travail. Les études de Sizaya et al, Sabitu et al, et Bedi et al. corroborent nos observations [25–27]. Les risques liés à l'empoussiérage aux fibres de coton sont quasi permanents. Les problèmes de santé sont les affections pulmonaires, la fièvre du coton etc. Les broncho-pneumopathies et certaines réactions allergiques sont fréquentes. Nos résultats corroborés par ceux de la littérature montrent que les symptômes respiratoires sont plus élevés chez les sujets exposés au coton. Les études faites en Tunisie [21] et en Egypte [22] et ailleurs [25, 28, 31] soulignent que les affections respiratoires restent plus élevées dans les usines textiles en occurrence la toux, la dyspnée et la douleur thoracique. La byssinose, une pneumopathie spécifique à l'exposition au coton survient souvent à long terme. Cette longue durée d'exposition a été également évoquée par plusieurs autres études dans le secteur textile [29, 32–34]. Les facteurs de risques physiques constatés sont non négligeables. Les mêmes observations ont été faites par les études d'Amri C et al. et de Mahmoud MT et al. [23, 24] faites au Maroc en Egypte, de même que celle d'Osibogun faite au Nigéria [35]. Les facteurs de risques chimiques concernent des expositions aux différents produits au niveau de delintage, de la teinture et de l'impression. Les études faits au Maroc et en Egypte [24, 25] restent explicites à ce sujet. Les facteurs liés à l'organisation du travail et à l'environnement du milieu de travail sont fréquents. Ils sont souvent consécutifs à la charge du travail, aux rythmes et horaires du travail, à l'insuffisance d'infrastructure, d’équipements du travail, et de moyen de protection, à l'absence de formation et/ou d'information, etc. Plusieurs études faites dans le secteur textile ont fait mention des mêmes observations que ceux relevées dans notre analyse de la situation du travail [23, 27, 29, 33].

Les risques environnementaux liés à l'industrie textile ont été également signalés. Les préoccupations qui se font jour au sujet de l'environnement dans l'industrie textile peuvent avoir deux origines à savoir les opérations de fabrication elles-mêmes et les risques liés au mode d'utilisation des produits [6, 11, 17]. Ces observations ont été également relayées par d'autres études [23–25]. Outre la toxicité éventuelle des substances, les odeurs désagréables posent souvent problème, notamment lorsque des ateliers de teinture et d'impression sont situés à proximité de zones résidentielles. Il ressort de la majorité d’études [11, 13, 14] que l'usage des grandes quantités d'intrants agricoles, engrais et produits phytosanitaires dans la culture du coton constitue une principale source d’écotoxicité. La contamination des eaux usées par les colorants non fixés pose un problème d'environnement grave, non seulement en raison des risques potentiels pour la santé de l’être humain et des animaux, mais aussi en raison de la forte visibilité des colorations produites. La pollution atmosphérique par la poussière, la fumée et autres déchets industriels est quasi permanente. Plusieurs autres études faites dans les textiles laissant ressortir ces observations [11, 17, 18]. Les pesticides peuvent agir au niveau de la population d'un écosystème, mais également au niveau de la structure et du fonctionnement de l’écosystème tout entier. Les effets néfastes non observables à court terme sont décrits dans la littérature [11–19]. Notons afin qu’à tous ces facteurs de risques professionnels, s'ajoutent les facteurs intrinsèques pouvant influencer négativement la santé des travailleurs; notamment l'hypertension artérielle, le diabète, le paludisme, la malnutrition, le tabagisme, l'alcoolisme, troubles de vision etc.), facteurs qui doivent être pris en compte dans l’évaluation des risques [2, 22]. A cela, il faudra aussi ajouter dans le contexte de la RDC, les conséquences multiples entraînées par les guerres successives d'agression dans cette région depuis environ une décennie.

**Limites du travail:** nous terminerons la discussion en parlant de biais potentiels pouvant influencer nos observations. Le biais de « healthly worker effect » souvent présent dans les études portant sur le milieu de travail est minimisé voire inexistant du fait que nos observations portent sur une population de travailleurs appartenant à une seule entreprise. La principale limite de ce travail tient lieu du fait que l’étude n'a porté que sur des données disponibles d'une seule entreprise parmi les trois fonctionnelle que compte le pays et est donc sujette à un biais de sélection dont l'effet sur les estimations est difficilement appréciable. Ce biais peut remettre en cause l'inférence de nos résultats à la population des travailleurs textiles du pays, compte tenu notamment de la situation socio-géographique particulière de la région.

## Conclusion

En RDC, le domaine de l'industrie textile est exclusivement représenté par le coton. Les facteurs de risques professionnels sont de natures diverses (physiques, chimiques, psychosociaux etc.) et quasi permanents. Les problèmes de sécurité et de santé au travail restent dominés par les affections liées aux pesticides, les affections respiratoires, les troubles musculosquelettiques, les affections liées aux bruits et aux vibrations, les lésions corporelles dues aux accidents du travail, les risques psychosociaux ainsi les problèmes liés à l'environnement. Les actions de prévention et protection des travailleurs associées à celles de la promotion de la sécurité et santé au travail doivent être envisagées. Les études plus approfondies et quantifiables sont à envisager pour relever les problèmes de sécurité et santé dans cette entreprise.
